# Sex Differences in the Association Between Testosterone and Violent Behaviors

**DOI:** 10.5812/traumamon.18040

**Published:** 2014-08-01

**Authors:** Shervin Assari, Cleopatra H. Caldwell, Marc A. Zimmerman

**Affiliations:** 1 Department of Health Behavior and Health Education, School of Public Health, University of Michigan, Michigan, United States; 2 Center for Research on Ethnicity, Culture and Health, School of Public Health, University of Michigan, Michigan, United States; 3 Michigan Youth Violence Prevention Center, School of Public Health, University of Michigan, Michigan, United States

**Keywords:** Testosterone, Young Adult, Sex

## Abstract

**Background:**

Research on the association between testosterone and violent behavior has provided conflicting findings. The majority of studies on the association between testosterone and antisocial-violent behaviors has used a clinical sample of severely violent individuals. These studies have mostly assessed males.

**Objectives:**

To study sex differences in the association between testosterone and violent behaviors in a community sample of young adults in the United States.

**Patients and Methods:**

A longitudinal study of an inner city population on subjects aged from adolescence to adulthood was undertaken. Testosterone and violent behaviors were measured among 257 young adults with an average age of 22 years (range 21 to 23 years). We used regression analysis to test the association between testosterone and violent behaviors in male and female samples.

**Results:**

There was a significant positive correlation between testosterone levels and violent behaviors among females, but not males. The association between testosterone levels and violent behaviors among females was significant, as it was above and beyond the effects of socio-economic status, age, education, and race.

**Conclusions:**

Our findings provide more information about the biological mechanisms for violent behaviors among young female adults. The study also helps us better understand sex differences in factors associated with violent behaviors in the community.

## 1. Background

Interpersonal violence is a serious public health problem in the United States and worldwide (1). Interpersonal violence may result in morbidity or death. Aggressive behaviors peak in early adulthood and adolescence (1-3), with up to one third engaging in an altercation each year (1). Due to the high rates of violence during adolescence and early adulthood, homicide is the second leading cause of death among individuals aged 15 to 24 years (3). Based on a report by the Centers for Disease Control and Prevention, in 2007 alone, interpersonal violence among adolescents and young adults resulted in 600,000 emergency department visits (3).

Although social and environmental risk factors for interpersonal violence are well known (4), the literature is not conclusive on the biological predictors (5). Testosterone is one of the most commonly studied biological markers that may be linked to aggressive behaviors. High testosterone levels have been associated with childhood conduct disorder and adulthood antisocial personality disorder (6-9). Testosterone levels may also be higher among children and adolescents with externalizing and delinquent behaviors, as well as among inmate adults (10-13).

As the literature has provided mixed results in this area (5), researchers have shown interest in finding possible moderators that can change the association between testosterone and aggression (5). In this regard, multiple factors such as gender, sex, socio-economic status, age, and past experience have been suggested as possible moderators. However, measurement, metabolism, and/or circadian rhythms of these biological markers may also contribute to these inconsistencies (5). Although sex and gender have been suggested as possible moderators of the link between testosterone and aggression, most studies have focused exclusively on males (14, 15). Furthermore, only a handful of studies have also enrolled females (16, 17). In addition, very few studies, if any, have tested such associations among a sample that is mostly composed of black subjects. Finally, most studies on the association between testosterone and violent behaviors have been conducted in a clinical setting on individuals with severe violent behaviors. Thus, more research is needed to understand the role of sex as a possible moderator for the association between testosterone and violent behaviors.

## 2. Objectives

In this study we investigated the moderating effect of sex on the association between testosterone and violent behaviors in a community sample of young adults in the United States.

## 3. Patients and Methods

Data came from the Flint Adolescent Study (FAS), a longitudinal cohort of an inner city population from 1994 to 2012. Data were collected anonymously. All participants provided consent forms before each interview. The University of Michigan Institutional Review Board approved the study protocol. Participants were enrolled from four local public high schools. The study enrolled students in the fall semester of the ninth grade if they had a grade point average (GPA) of 3.0 or lower in eighth-grade, and they did not have a diagnosis of developmental disability or emotional impairment. The FAS enrolled 850 adolescents from the ninth grade and followed them through their transition into adulthood. The current analysis only included participants who provided saliva samples in Wave 6. Thus, the current study only included a subset of participants (n = 257) who had participated in Wave 6 in the year 2000 (n = 573) of the study, and who met the following eligibility criteria for collecting saliva samples. Eligibility for saliva collection included; providing consent for the procedure, not being pregnant, not having anything to eat, drinking nothing except water, and not using tobacco, 1 hour prior to collection.

Youth who consented to saliva sampling were not different from the overall group of Wave 6 participants. The study has 12 Waves of data and detailed data on demographics (e.g. age and sex), socio-economics (e.g. family structure and parent employment), psychological factors (e.g. coping and impulse control), family relations (e.g. family closeness and relations with parents), religion (e.g. frequency of church attendance), social relations (e.g. relations with friends), behaviors (e.g. violence and substance use), and health (body mass index and perceived health). Participants were between 14 and 17 years of age in the first Wave, with a mean age of 15 years. Retention rates were 90% from Waves 1 to 4 and 75% from Waves 4 to 8.

### 3.1. Procedure

Data were collected during structured face-to-face interviews conducted either at school or at alternative community locations. Interviews were carried out using university-trained members of our research team. On average, each interview lasted 50–60 minutes. A self-administered questionnaire assessed more sensitive information and this was distributed at the conclusion of each interview to facilitate confidentiality.

Covariates: Age and socio-economic status (i.e. education) were used as control variables in the multivariate analysis. Age was a continuous variable. Education was operationalized as a continuous variable, measured as highest level of education.

Violent behaviors: Violent behaviors were measured using the following seven items. Youths were asked how often they had engaged in the following behaviors; 'had a fight in school', 'taken part in a rumble where a group of your friends were against another group', injured someone badly enough to need bandages or a doctor', 'hit a teacher or supervisor (work supervisor)', 'used a knife or gun or other object (like a club) to get something from a person', 'carried a knife or razor', or 'carried a gun'. All items used a Likert response, ranging from 1 (0 times) to 5 (4 or more times). Responses to each item were averaged to calculate the behavior during the past year. Total score was calculated as the average of all items. Higher scores indicated more violent behaviors (α = 0.79). This measure has shown high reliability and validity and it has been used previously in several published reports (18, 19).

Testosterone: Testosterone was measured at Wave 6, using saliva samples. Participants were asked to collect saliva in their mouths for one minute, and then spit slowly into a cryotube. Following all consent procedures, respondents rinsed their mouths with water. All samples were collected after 11:00 a.m. to control for changes due to diurnal rhythm. The saliva samples were placed on ice and refrigerated until transportation. They were kept at -80°F for storage. Saliva was collected only from participants who were not pregnant, had not smoked, or ingested anything other than water in the hour prior to testing. Saliva samples with a blood protein contamination greater than 3 mg/dL were excluded (20).

Testosterone levels were assessed by a high sensitivity salivary cortisol enzyme immunoassay (Salimetrics, Inc., PA, United States) (20). The saliva samples were thawed and centrifuged at 1500 rpm for 15 minutes before assay. The assay followed standard enzyme immunoassay procedures as described elsewhere (20).

Data analysis: Data analysis was conducted using SPSS 20.0 (IBM Inc., IL, USA) for Windows. Means and standard deviations (SD) were reported for age, testosterone, and violent behaviors for both males and females. Pearson’s correlation test was used to measure bivariate associations between the study variables. We fitted linear regression models (method = enter) to test the association between testosterone and violent behaviors. The dependent variable was aggressive behaviors, the predictor was testosterone, and age, education and race were controls. We fitted models to the pooled sample, and also among each sex. P values less than 0.05 were considered significant.

## 4. Results

### 4.1. Descriptive Statistics

Most of the participants (86.4%) were black or mixed race, and only a minority (13.6%) were white. Most participants had some college education (41.2%) and were living with a partner or relative (58.4%). Only a minority of the participants (21.4%) were unemployed (Table 1).

The mean age was 20.5 years at Wave 6 in both male and female subjects (SD = 7). Testosterone and aggressive behaviors were higher among the male than the female participants (Table 2).

**Table 1.** Socio-Economic Status Among Male and Female Young Adults

	All	Men	Women
**Employment, No. (%)**			
Working	111 (43.2)	63 (50.4)	163 (36.4)
Work/student	60 (23.3)	22 (17.6)	38 (28.8)
Student	30 (11.7)	13 (10.4)	17 (12.9)
Unemployed	55 (21.4)	26 (20.8)	29 (22.0)
**Education, No. (%)**			
No degree	56 (21.8)	34 (27.2)	22 (16.7)
General Educational Development or High School Diploma	95 (37.0)	52 (41.6)	43 (32.6)
Some college	106 (41.2)	39 (31.2)	67 (50.8)
**Living status, No. (%)**			
With parents/relatives	150 (58.4)	84 (67.2)	66 (50.0)
Alone	106 (41.2)	40 (32.0)	66 (50.0)
**Race, No. (%)**			
White	35 (13.6)	16 (12.8)	19 (14.4)
Black/Mixed Race	222 (86.4)	109 (87.2)	113 (85.6)

**Table 2.** Descriptive Data Relative to Age, Aggressive Behaviors and Testosterone Among Men and Women^a^

	All	Males	Females	P value
**Age, y**	20.52 ± 0.64	20.58 ± 0.68	20.47 ± 0.61	0.186
**Aggressive behaviors**	1.27 ± 0.48	1.38 ± 0.58	1.16 ± 0.33	< 0.001
**Testosterone, pg/mL**	97.27 ± 88.71	150.89 ± 95.40	46.50 ± 37.73	< 0.001

^a^ Data are presented as Mean ± SD

### 4.2. Bivariate Correlations

Among the selected factors, testosterone and aggressive behaviors were positively correlated, while education was negatively correlated with testosterone and aggressive behaviors. Age was associated with education, but not testosterone or aggressive behaviors. Race was not associated with age, education, testosterone or aggressive behaviors among men or women. Education was associated with aggressive behaviors among men, but not women (Table 3).

As Table 3 suggests, there was a correlation between testosterone and aggressive behaviors only among the females. Age was associated with aggressive behaviors among women, while education was associated with aggressive behaviors among men.

**Table 3.** Correlation Matrix Between Socio-Economics, Testosterone and Aggressive Behaviors Among Male and Young Female Adults ^a^

	1	2	3	4	5
**All**					
1 Age, y	1	-0.33	0.06	0.1	0.1
2 Education		1	0.01	-0.20	-0.24
3 Black			1	0.01	0.04
4 Testosterone, pg/mL				1	0.16
5 Aggressive behaviors					1
**Males**					
1 Age, y	1	-0.29	0.04	0.08	0.008
2 Education		1	-0.04	-0.17	-0.27
3 Black			1	0.002	0.12
4 Testosterone, pg/mL				1	-0.004
5 Aggressive behaviors					1
**Females**					
1 Age, y	1	-0.35	0.07	0.03	0.22
2 Education		1	0.07	0.03	-0.10
3 Black			1	-0.01	-0.08
4 Testosterone, pg/mL				1	0.19
5 Aggressive behaviors					1

^a^All correlation coefficients larger than 0.15 are statistically significant at P <0.05.

### 4.3. Regression Analysis

As Table 4 suggests, in the pooled sample, sex and education were associated with aggressive behaviors. In the pooled sample, female sex and high levels of education were associated with low levels of aggressive behaviors. Our model in the male sample suggested that males with higher levels of education had lower aggressive behaviors. Among males, testosterone was not associated with aggressive behaviors. Among women, however, testosterone was positively associated with aggressive behaviors. Women with higher levels of education had lower levels of aggressive behaviors (Table 4).

**Table 4.** Summary of Regression Testing the Association Between Testosterone and Aggressive Behaviors Among Male and Female Young Adults

	95.0% Confidence Intervals for Bounds			
	Standardized Beta	Lower Bound	Upper Bound	P value
**All Participants**				
Age, y	0.01	-0.08	0.10	0.86
Black	0.04	-0.11	0.22	0.49
Female	-0.18	-0.31	-0.03	0.02
Education	-0.20	-0.20	-0.04	0.002
Testosterone, pg/mL	0.01	-0.001	0.001	0.86
**Males**				
Age, y	-0.07	-0.21	0.09	0.44
Black	0.11	-0.10	0.49	0.20
Education	-0.30	-0.36	-0.09	0.001
Testosterone, pg/mL	-0.05	-0.001	0.001	0.57
**Females**				
Age, y	0.20	0.007	0.20	0.04
Black	-0.10	-0.25	0.07	0.26
Education	-0.03	-0.10	0.07	0.74
Testosterone, pg/mL	0.18	0.001	0.003	0.04

## 5. Discussion

The current study tested the association between testosterone and violent behaviors in a community sample of male and female young adults in the United States. The study was conducted in response to the gap in knowledge about sex differences in the association between testosterone and violent behaviors at the community level. Our findings revealed a positive correlation between the level of testosterone and violent behaviors among young female, but not among young male adults. Thus, the results suggested heterogeneity of the testosterone-violence link based on sex. The contribution of the current study is significant because most current literature is composed of studies conducted in clinical settings. Such studies have enrolled individuals with psychiatric disorders (6, 11), criminals, rapists, or prisoners (8, 11). Small sample size is also a limitation of many of these studies (7, 8, 13, 21), and a majority of the studies are restricted to male participants (6-9). In addition, almost all studies in the field have used a cross-sectional design. Thus, the findings of the current study may potentially help us increase our understanding of sex differences in biological mechanisms for violent behavior in a community sample.

In line with some previous studies (15), our study did not show an association between testosterone and violent behaviors among men. A study on the links between testosterone, aggressiveness/hostility, and antisocial personality published in 1999 showed that violent and nonviolent men did not differ in total plasma testosterone levels on any sampling occasion. Among violent men, however, testosterone levels had a positive and significant association with hostility (7). There are, however, studies suggesting that high testosterone levels in cerebrospinal fluid, serum, and saliva may predict aggressive behavior (22), violent crime (14, 15), and antisocial personality disorder (23), among men. Testosterone levels may also predict social presentation of masculinity and toughness (14), among males.

Our study documented a link between testosterone and aggressive behaviors among young female adults who lived in the inner city, while the effect of age, race, and education was controlled. One study conducted among female adolescents has suggested that high levels of testosterone may predict conduct disorder (17). On the other hand, there are studies that failed to show such associations with externalizing behaviors (17).

Results of previous studies on the association between testosterone and aggression are therefore not conclusive. While some of the previous studies have reported a positive relationship between testosterone and aggressive behaviors, there are other studies reporting a negative or non-association between levels of testosterone and aggression (5, 24).

Human studies have suggested that higher testosterone levels may be linked to aggression, social dominance, and hyper-reactivity to status threats (25, 26). Interestingly, individuals with psychiatric disorders that present impulsive aggression (e.g. antisocial personality disorder and borderline personality disorder) may have higher levels of testosterone (6, 27, 28). Some researchers believe that it does not affect all forms of aggression, and that only those related to impulsive aggression in response to social threat are associated with testosterone (29).

There are, however, studies suggesting that the strength and direction of the association between testosterone and aggression may not be different among men and women (30). Animal studies suggest that injection of androgens may result in increased aggressive behaviors among female primates (31). Studies among humans have also suggested that androgen therapy may increase anger among women (32). Another observational study also documented positive correlations between testosterone and aggression during early follicular and late luteal phases of menses (33). These factors may also explain why sex differences may exist in the link between testosterone and aggression. Future research should test the role of menstrual cycle phases as a possible moderator as well.

The current study suggested that sex may moderate the link between testosterone and aggressive behaviors among young adults who live in urban areas. Another study measured plasma levels of testosterone and also externalizing behaviors among 51 boys and 68 girls at age 14. The study used the Achenbach Child Behavior Checklist and the Youth Self Report Form to assess externalizing behaviors. Only among males was there a positive correlation between androgen metabolites and externalizing problems. Such an association could not be found among women (12). In contrast, another study suggested a link between testosterone and externalizing behavior in male adolescents, but not females. In this study testosterone was measured among 87, 14-year-old adolescents (36 boys, 51 girls), and externalizing behavior was measured at age 8, 11 and 14 years. Results suggested that plasma testosterone predicted persistent externalizing behavior among males, but not females (13).

Age may explain some of the conflicting results across the studies. A meta-analysis of community and selected samples suggested that there might be only low to modest association between testosterone and aggression, with mean weighted correlations ranging from 0.08 to 0.14, in males. Overall, these meta-analyses suggest that the testosterone-aggression association is equally strong in 12 to 21-year-olds, as it is in 22 to 35-year-olds, but that it may be less strong in age groups younger than 12, than in those who are older (5, 34, 35).

The results of the current study were in contrast with the literature which suggests that a link between testosterone and violent behaviors exists among male, but not female individuals. One of the many factors that may explain the inconsistency in these findings is the community versus clinical setting, which has been shown to be a determinant of these associations. Literature has previously shown that many of the findings that can be found in clinical samples may not be easily replicated in a community setting (36).

Previous researchers have proposed mechanisms to explain sex differences in the link between testosterone and aggressive behaviors (24). One possible reason is that women have lower levels of testosterone and aggressiveness compared to men (24). One study found a rise in testosterone levels prior to a contest among men, but not women (29). It has been suggested that the effect of competition (mostly aggressive behaviors) on increasing testosterone levels may only be present among men (29).

It is also plausible to attribute sex differences in the above studies to differential variations in the amount of testosterone among men and women. Such variations may attenuate the observed correlations in one sex (5). For instance, those studies that show a smaller relationship between testosterone and aggression among females may be due to range restriction (5). However, we can argue that just because testosterone and aggressive behaviors are lower among females, it does not mean that the relationship does not exist among women. In this view, higher levels of testosterone lead to more aggressive behavior, irrespective of the fact that the range may be more limited among women. Finally, it may be easier to obtain accurate testosterone measurements in females, due to less diurnal variation, a phenomenon that is in contrast with the expected range restriction among females (5).

The current study may also have public health implications. The aggressive behaviors observed in women who live in a very violent community may be under the influence of testosterone. Interestingly, the same finding could not be found among men. The study also suggested a role of education level on the aggressive behaviors of men. Thus, we may argue that aggressive behaviors may be more social and less biologically based among men. As social factors can be modified, and biological factors are less subject to change, interventions for the prevention of violence may be easier for men. Future research should test the complex interactions between sex, gender, biology, and social environment in shaping the aggressive behaviors of residents living in violent communities.
